# Supplementary figures and images for: Genetic origins and proteomic consequences of kinetoplast loss in trypanosomes
Source: PLoS Pathog. 2026 Mar 25;22(3):e1013846. doi: 10.1371/journal.ppat.1013846 (PMC13035230; doi:10.1371/journal.ppat.1013846)

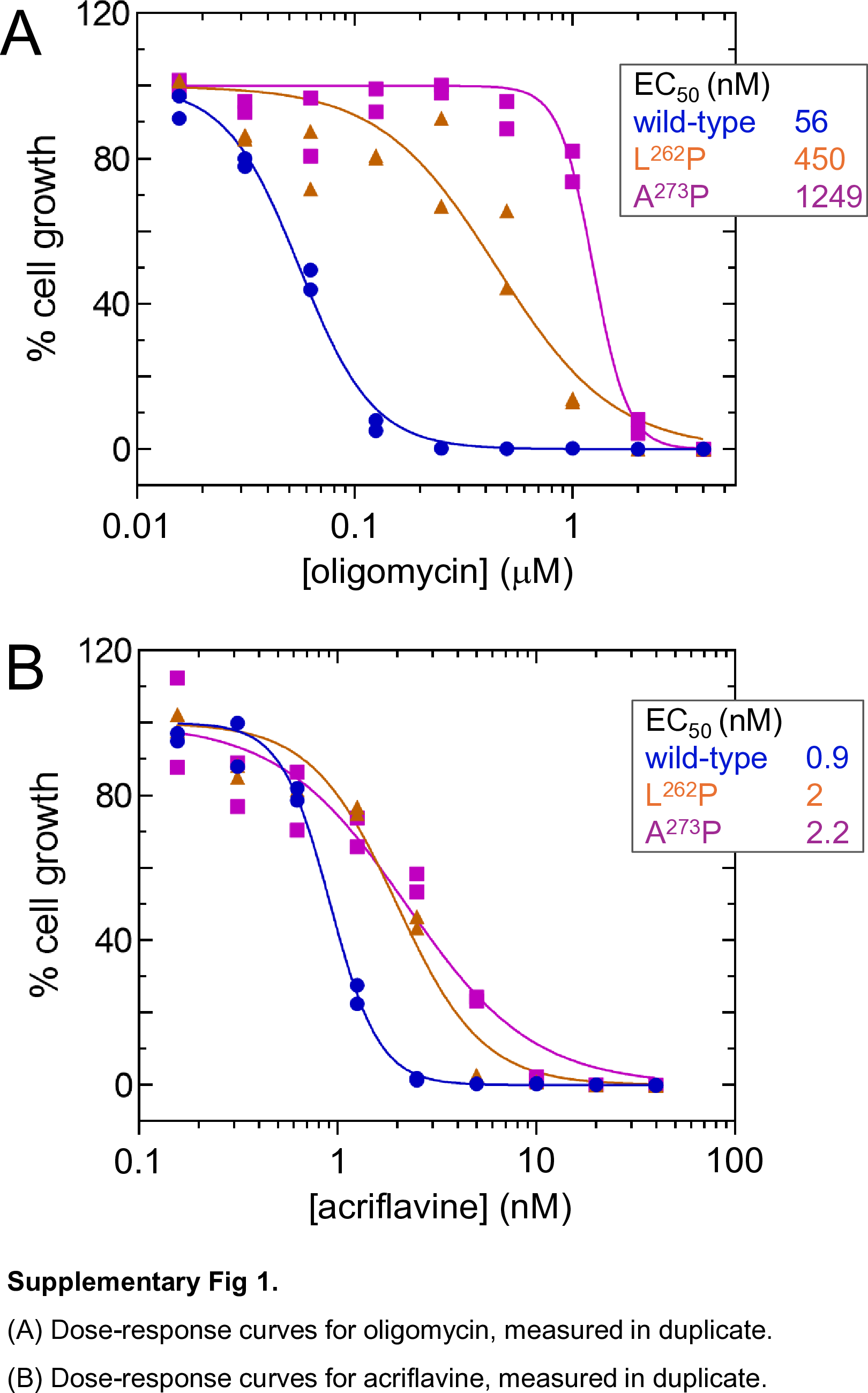

Supplement: S1 Fig — For oligomycin, measured in duplicate. (B) For acriflavine, measured in duplicate. (TIF) [file ppat.1013846.s001.tif]

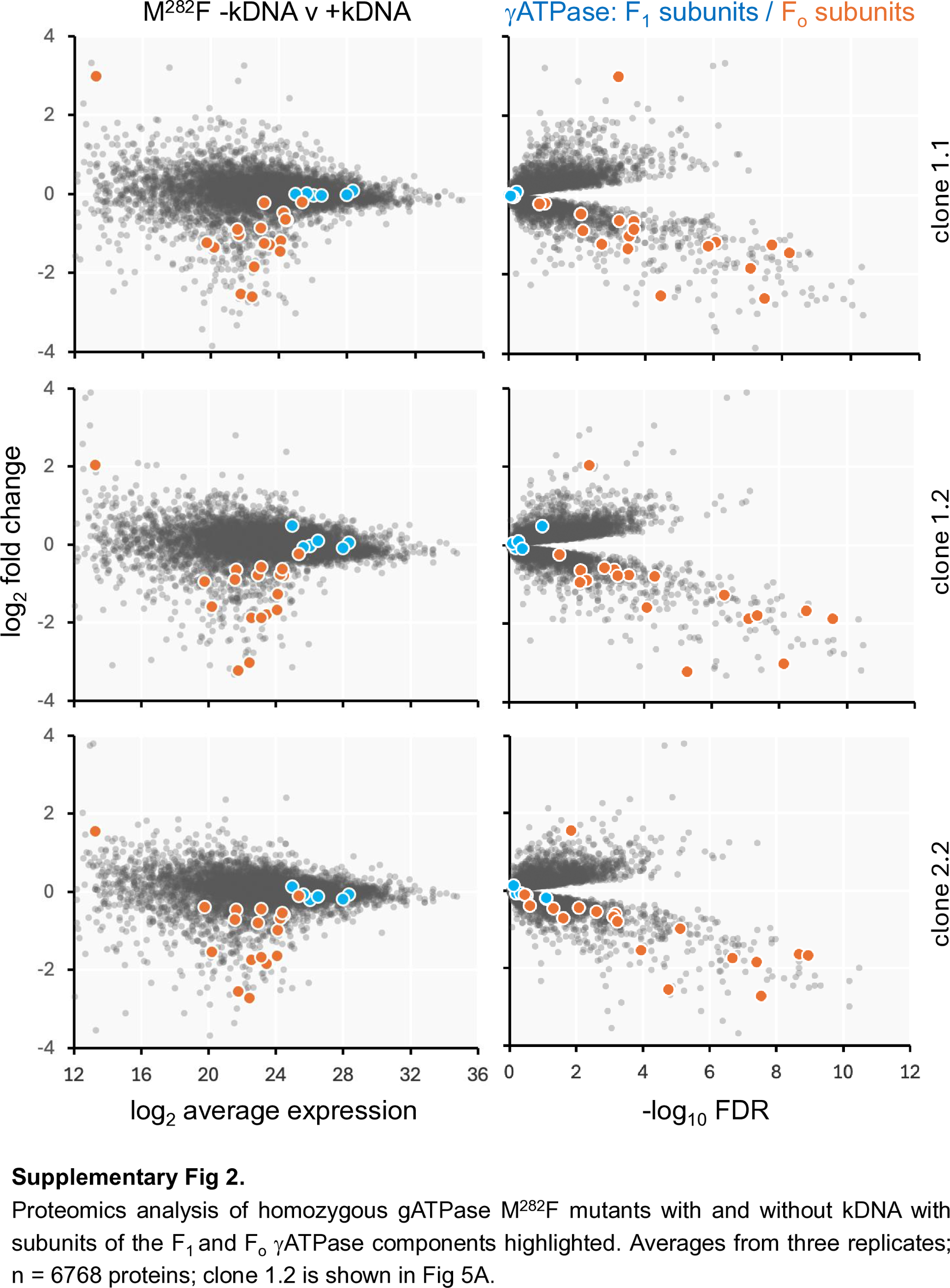

Supplement: S2 Fig — Subunits of the F1 and Fo γATPase components are highlighted. Averages from three replicates; n = 6768 proteins; clone 1.2 is shown in Fig 5A. (TIF) [file ppat.1013846.s002.tif]

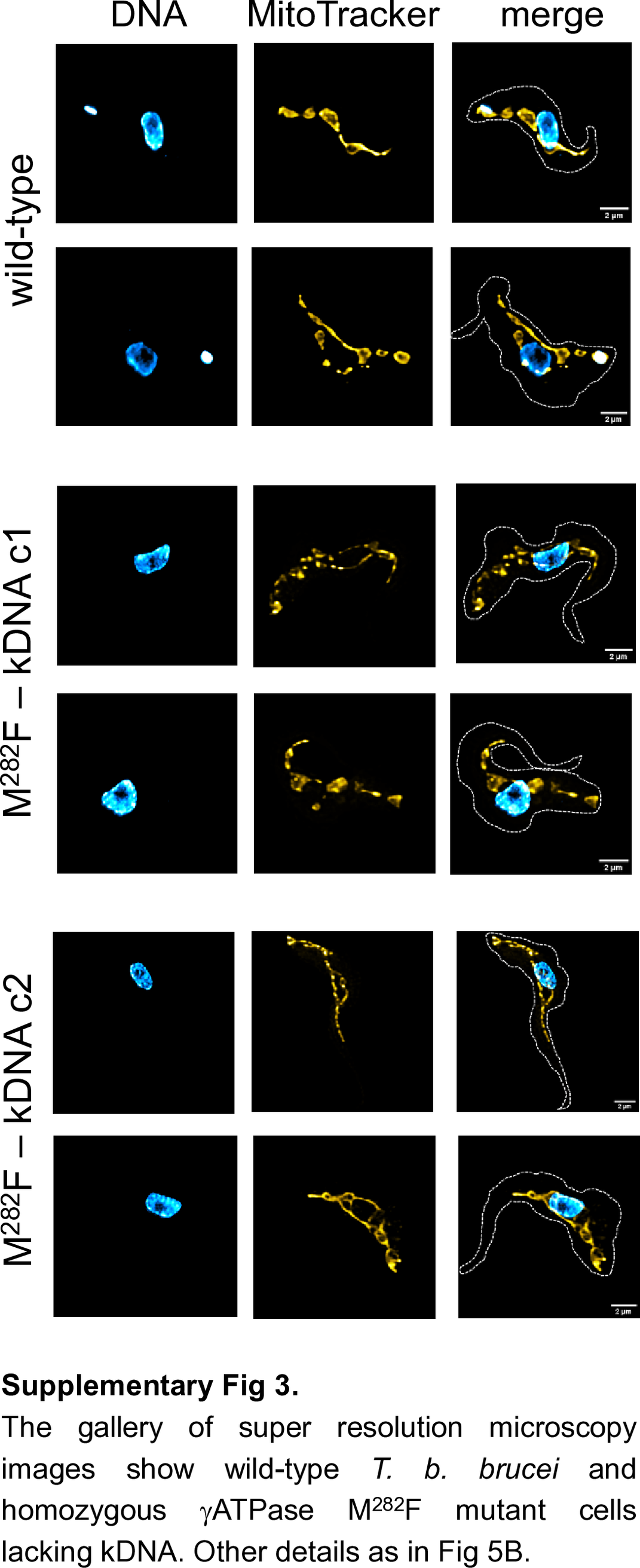

Supplement: S3 Fig — Other details as in Fig 5B. (TIF) [file ppat.1013846.s003.tif]
