# Supplementary material for: Genetic origins and proteomic consequences of kinetoplast loss in trypanosomes
Source: PLoS Pathog. 2026 Mar 25;22(3):e1013846. doi: 10.1371/journal.ppat.1013846 (PMC13035230; doi:10.1371/journal.ppat.1013846)
Supplement: Attachment — Submitted filename: Ridgway 03269 Reviewer responses.pdf [file ppat.1013846.s006.pdf]

PPATHOGENS-D-25-03269
Genetic origins and proteomic consequences of kinetoplast loss in trypanosomes

Reviewers' Comments:

Part I – Summary

Reviewer 1:
This manuscript investigates how mutations in the gamma subunit of the mitochondrial F_1F_0-ATP synthase (gATPase) facilitate survival of Trypanosoma brucei in the absence of the kinetoplast DNA (kDNA). The study builds on the observation that certain gATPase mutations render T. brucei resistant to drugs targeting mitochondrial functions and enable life without kDNA. By employing targeted gene editing and selection with the ATP synthase inhibitor oligomycin, the authors isolate and characterize novel and known gATPase mutations (M282F, M282W, M282Y, L262P, A273P) and analyze their biochemical, proteomic, and cellular consequences.
This work provides an important contribution to our understanding of mitochondrial genome independence in eukaryotes and explores the functional consequences of mitochondrial genome loss, which has implications for parasite biology, transmission, and drug resistance. However, some aspects of the mechanistic interpretation and data presentation require further clarification and experimental support.

Major Claims by the Authors
1. Mutations in the gamma subunit enable viability without kDNA including the newly found M282F homozygous mutant
2. Study aims to explore the origins and consequences of kDNA loss
3. Use of oligomycin for enrichment of gamma ATPase mutants following oligo-directed mutagenesis
4. Homozygous M282F mutants resist acriflavine and survive kDNA loss
5. Proteomic analyses show mitochondrial remodeling associated with kDNA loss
o Pre-kDNA loss: Specific depletion of ATP synthase-associated proteins (excluding F_1 subunits).
o Post-kDNA loss: Loss of kDNA-binding proteins and mitochondrial RNA processing factors; increase in mitochondrial membrane-associated transporters.
6. Conclusion
o Cells with homozygous M282F mutations assemble a remodeled ATP synthase, tolerate kDNA loss, and undergo substantial mitochondrial proteome remodeling.

Reviewer 2:
While the flexible mitochondrial biology of Trypanosoma brucei has been studied in detail, knowledge about how mutations that render the cells susceptible to kDNA loss and their full impact during drug treatments are understudied. In this body of work, the authors use a rapid and specific precision editing tool based upon oligo-targeting first reported in the field by the Horn lab. They combined this with a selection using 200 nm oligomycin. The authors generated mutants against known gamma ATPase mutations associated with kDNA dispensability and recover new mutations at the position M282. The methodology is robust with good internal controls to distinguish heterozygote and homozygous mutants. The authors are laying the foundational tools for futures studies on analyzing mutations that occur in the wild and could drive drug resistance. There is a lot of interesting data that was generated but perhaps not fully analyzed. The heterogyzous mutant seems important for the trajectory of the population becoming kDNA dispenable but was not fully analyzed.

Reviewer 3:
This study demonstrates that specific mutations in the nuclear-encoded γ subunit of the mitochondrial F_1F_0-ATP synthase enable Trypanosoma brucei to tolerate complete loss of

kinetoplast DNA. Using precision oligo-targeting, the authors identify a novel homozygous γATPase M282F mutation that confers reduced sensitivity to both oligomycin and the kDNA-targeting drug acriflavine, and show that this mutation permits acriflavine-induced elimination of the mitochondrial genome. Quantitative proteomics reveals that γATPase mutation leads to selective remodelling of the ATP synthase complex prior to kDNA loss, followed by extensive mitochondrial proteome reprogramming after kDNA loss, including depletion of kDNA-binding and RNA-processing factors and upregulation of mitochondrial membrane transport systems. While the study does not provide fundamentally new mechanistic insight into how γATPase mutations uncouple the ATP synthase, the proteomic analyses generate a valuable dataset that may be useful for further exploration of cellular adaptations to mitochondrial DNA loss.

Part II – Major Issues: Key Experiments Required for Acceptance

Reviewer 1:
1.1: Oligomycin Resistance and Wild-Type gamma ATPase Contribution
The manuscript should explore whether the level of oligomycin resistance correlates with residual wild-type gamma ATPase expression in heterozygous strains. Such analysis would provide insights into the dosage sensitivity and functional threshold of the mutant protein.
R1.1: We had carefully considered more detailed analysis of heterozygotes, but as stated in our Results section "Since differential expression of mutant alleles could impact the behaviour of heterozygous mutants, we favoured the analysis of homozygous mutants". We also note that "both L^{262}P and A^{273}P γATPase substitutions with a validated link to kDNA dispensability are present as homozygous mutations in *T. b. brucei* [12] and *T. b. equiperdum* [14], respectively". A "heterozygous $A^{281}\Delta$ mutant γATPase allele was reported to be preferentially expressed in *T. b. evansi* [14]", but this was based on mRNA abundance, which of course may not reflect relative protein abundance. Even if we were able to determine the relative levels of wild-type and mutant γATPase protein in the heterozygous strains, we would be unable to determine relative levels in individual cells, which could impact oligomycin resistance and other phenotypes.

1.2: Mitochondrial Morphology and Organelle Interaction
How do these mutations affect:
• Mitochondrial ultrastructure?
• ER-mitochondria contact sites?
• Electrochemical potential across the mitochondrial membrane?
These aspects are central to mitochondrial function and may be altered in kDNA-lacking cells. The authors should consider EM or confocal imaging, and membrane potential assays.
R1.2: We used a "Leica Stellaris 8 inverted confocal microscope". We have also now conducted a MitoTracker staining and flow cytometry assay and include these data in Fig. 3C and Fig. 5C. We now also describe "DNA and MitoTracker staining followed by super resolution microscopy…" and have added a gallery of super-resolution images in Supplementary Fig. 3.

1.3: ATP Synthase Assembly Pathway
The authors suggest that assembly is arrested post-F_1–c-ring attachment, in analogy to other eukaryotes. This hypothesis is testable using blue native PAGE. Inclusion of such data or acknowledgment of this as a future direction would strengthen mechanistic claims.
R1.3: We've added "…; a hypothesis that could be tested in the future" here.

1.4: TAC Components and kDNA Loss
Given the complete loss of kDNA in homozygous mutants, the status of TAC (tripartite attachment complex) proteins should be addressed. Are these proteins depleted? Their

inclusion in the proteomic analysis (especially in Figure 5) would provide a useful internal control and functional insight.

R1.4: In general, and since the full dataset is included in the Supplementary Data 1 Excel file, we decided not to discuss cohorts or complexes in the main text that fail to register highly significant changes, or individual proteins that register < 2-fold change. We have made an exception in this case though, and now state, "The tripartite attachment complex itself (Jetishi *et al.*, 2025) is notably not substantially depleted, and only the p166 component (Tb927.11.3290) achieved an FDR <0.01 following kDNA loss (log2 fold-change = -0.52 +/- 0.12); indeed, kDNA is not required for assembly of this complex (Hoffmann *et al.*, 2018).". We also now highlight proteins involved in "kDNA anchoring to the tripartite attachment complex (Amodeo *et al.*, 2023)" and identify both KAP68 and mtHMG44 in Fig. 6B.

1.5: Mitochondrial Import Machinery

The increase in membrane-associated transporters is noted, but specific components and the extent of change should be detailed in the main text (not solely in figures). Which import components are increased, and by how much?

R1.5: Figure 5E shows data for 74 MCP / TIM / ATOM / MICOS / POMP proteins among many others. Readers may also be interested in other "proteins that were significantly (FDR <0.01) reduced (n = 507) or increased (n = 761) in abundance following kDNA loss". Rather than detailing further specific proteins in the main text, we prefer to refer readers to "(Supplementary Data 1)" where the full dataset can be explored and mined.

Figure-Specific Critiques

1.6: Figure 1D

• Structural labeling is incomplete. Clearly label F_1 and F_0 domains.

R1.6a: We've added details to the figure legend.

• Mutation positions (e.g., M282F) should be mapped explicitly also in the stick figure.

R1.6b: We feel that it could be misleading to attempt to map the mutations in the schematic. Also, subunit γ rotates relative to the α/β hexamer (as indicated in the schematic).

• Color coding lacks explanation; provide a legend or rationale.

R1.6c: These details have also now been added to the figure legend.

1.7: Figure 3

• Clarify the meaning of n in the legend; does not refer to replicates?

R1.7a: We've adjusted the legend: "n = 6564 proteins".

• Justify inclusion of both $\log_2$ fold-change and $-\log_{10}$ FDR. Are both dimensions necessary?

R1.7b: Log_2 fold-change captures magnitude of the effect while $-\log_{10}$ FDR reflects statistical confidence. Filtering on both means that the changes identified are biologically meaningful (fold-change) and statistically robust (FDR).

• Consider whether this figure is redundant with Figure 5. If retained, explain the complementary value.

R1.7c: This "proteomic analysis revealed highly specific depletion of subunits of the F_O component of the *T. b. brucei* ATP synthase in homozygous $M^{282}F^{TTT}$ mutants pre kDNA loss" while Figure 5 shows "Mitochondrial proteome remodelling following kDNA loss".

1.8: Figure 4A

• Discrepancy noted between DNA channel and merged image.

R1.8a: These panels are now shown in Fig. 5B. We've adjusted the panels to show loss of kDNA more clearly and also now outline the cells in the merged panels.

• A single-cell image is insufficient to claim kDNA loss. Include a field with multiple cells for representativeness.

R1.8b: Several cells are now shown in the panels in Fig. 4A. We also note that whole genome sequencing data provide an orthogonal, and in our view more robust assay that demonstrates kDNA loss.

1.9: Figure 5
• The presentation is inconsistent and cluttered. Multiple visualization types could/should be unified.
R1.9a: We've split Fig. 5 into two Figures, which we believe improves the format.
• Cohort terms (Figure 5E) require definition. 400+ proteins for "kinetoplast" are a lot? Is that real? Are functional overlaps considered, i.e. the ribosome etc.?
R1.9b: An excellent point. This kinetoplast GO-term annotated cohort is based on an artifact of mNG when in the mitochondrion. We now use the cohort of kinetoplast-associated proteins defined by Pyrih *et al.*, 2023 (n = 58).
• Indicate what red/blue colors represent.
R1.9c: We've added "Data are shown for kDNA negative clones 1.2 (blue) and 2.1 (red)".
• Include TAC proteins to assess stability of mitochondrial structural elements.
R1.9d: See R1.4 above.
• Revise labeling of "increased" and "decreased" with fold enrichment to improve clarity.
R1.9e: The legend states proteins that are "decreased or increased in abundance in kDNA negative cells".
• PUF9 Target 1 Speculation: The discussion suggests that kDNA loss disrupts kDNA–nuclear genome communication. This needs clarification. What is the functional relevance of PUF9 targets in this context? How does their regulation support the proposed communication axis?
R1.9f: "Communication between the kDNA and nuclear DNA" via PUF9 and PNT1 was proposed previously, based on the work described in references 25 and 27. We propose that the changes in these proteins reflect disrupted communication and have now adjusted the text to "PUF9, involved in [nuclear] cell cycle regulation" which we hope helps to clarify.

1.10: Scope and Novelty: While the experiments are technically sound and well-conceived, the manuscript would benefit from a clearer articulation of what new insights this study adds over prior work. Specifically:
o What do the newly identified mutations (e.g., M282F) reveal beyond earlier L262P and A273P studies?
R1.10a: New insights are detailed in our abstract, briefly:

- Heterozygous and homozygous $M^{282}F$ mutants display different drug-resistance profiles.
- Proteomics analysis revealed ATP synthase remodelling pre-kDNA-loss.
- We further describe mitochondrial proteome remodelling post kDNA-loss.

We are not aware of any similar analyses using $L^{262}P$ or $A^{273}P$ mutants.
o How does this work advance our understanding of the origins (not just consequences) of kDNA loss?
R1.10b: See the paragraph beginning "In terms of the origins of kDNA loss…" in the Discussion, which describes genetic and proteomic changes that precede kDNA loss. We've also adjusted our title to be more specific, now "Genetic origins and proteomic consequences of kinetoplast loss in trypanosomes" (R3.1mi below).

1.11: Comparative Context: The authors may consider placing their findings in the broader context of mitochondrial genome loss in other systems (e.g., Saccharomyces cerevisiae "petite" mutants) to highlight evolutionary or mechanistic parallels.
R1.11: We do note that "Trypanosomes with γATPase mutations have emerged several times independently, being equivalent to mutations that enable mtDNA loss in petite-negative yeast [8, 14, 15]", but prefer to focus on implications for the trypanosomes in our Discussion.

Reviewer 2:
2.1: In the current study it appears that the kDNA loss does not occur until after drug pressure from acriflavine. This is not a compound that would be used in practice though. If one is considering what is driving kDNA loss and drug resistance in the field perhaps using a drug

more relevant to human treatments is warranted. With proof of principle in place with acriflavine, the authors should use a drug(s) in use and evaluate the outcome of their homozygous M282F mutant (perhaps even the hetrozygote).

R2.1: *T. equiperdum* and *T. evansi* were identified in the 1800's and had already spread beyond Africa before widespread use of kDNA-targeting therapies; we've added "first identified in the 1800's" to our Introduction. It is possible that kDNA targeting drugs select for γATPase mutants in the field but this is speculation. Our results do have the potential to be informative in this regard, but our study was not designed to understand "what is driving kDNA loss and drug resistance in the field". Rather, we wanted to understand γATPase mutations that render the kinetoplast dispensable and the consequences of kDNA loss. We used acriflavine as an experimental compound as we and others have previously used this compound to induce kDNA loss (see ref's 6 and 12 for example).

2.2: Authors should provide more details on the proteomics experiment of homozygous M282F mutant vs WT. There is clearly a subset of proteins other than F0 or F1 subunits that are also impacted when generating this mutant cell line. Did the authors perform proteomics on the M282F hetrozygote to identify significant changes form WT that were emerging to drive the population toward becoming kDNA dispensible? This progression would be then interesting to correlate with the different responses to acriflavine and oligomycin dose-response data.

R2.2: Eleven proteins achieved a $-\log_{10}$FDR >3 in the $M^{282}F$ homozygote, all depleted and all ATPase subunits. We checked those three proteins not highlighted as ATPase associated in our Figure panel and realised that they are indeed ATPase associated proteins; apologies for the error here and Fig. 3A and Fig. 5A have been adjusted accordingly. We did not perform proteomics on the $M^{282}F$ heterozygote as we don't consider it to have changes that 'drive the population toward becoming kDNA dispensable', as indicated by a similar acriflavine EC_{50} relative to wild-type cells (see Fig. 2C).

2.3: Similarly because selection of the ATPase mutants is already an experiment – the authors should report percent loss of kDNA compared to WT for each of the precision oligo edited clones that were generated (ie. prior to sub-lethal treatment with acriflavine or other drug).

R2.3: We previously stated "More than 99 % of these [homozygous $M^{282}F$] cells were kDNA positive prior to exposure to acriflavine" and now state "more than 99 % of heterozygous $L^{262}P$ and $A^{273}P$ mutant cells and homozygous $M^{282}F$ mutant cells remained kDNA positive by microscopy".

2.4: Could the authors please rewrite the abstract. The authors' summary was more informative/clear than the abstract (just lacked some specifics). For example in the abstract pre-kDNA-loss is introduced out of context; proposed a remodelled ATP suynthase complex – was this isolated and characterized? Rewriting would provide better clarity.

R2.4: We've adjusted the abstract and now use "kDNA-positive", "Proteomic analysis following acriflavine-induced kDNA loss" and "remodel ATP synthase subunit expression".

2.5: It would be beneficial if the authors could focus the discussion more on how homozygous vs heterozygous mutations could drive events in the wild. This is interesting and directly related to the problem of emerging subspecies and drug resistance….and related this to current drug classes that are used or overused in practice.

R2.5: We do say that "the widespread use of veterinary anti-trypanosomal drugs that target the kDNA, such as the DNA-damaging agents, ethidium bromide and isometamidium, could induce γATPase mutations and/or other mutations" in the Discussion. We've now added "… and promote kDNA loss in the field", but prefer not to speculate further on this point (also see R2.1 above).

Reviewer 3:

3.1: Although the genetic and proteomic analyses strongly suggest altered ATP synthase function and mitochondrial physiology in the gamma ATPase mutants, the study remains largely descriptive. The manuscript would be strengthened by direct functional measurements, such as mitochondrial membrane potential, ATP levels, or ATPase activity, before and after kDNA loss. Such data would more firmly link the observed molecular changes to mitochondrial function.
R3.1: We have now assessed MitoTracker staining before and after kDNA loss, which provides a measure of mitochondrial membrane potential (see Fig. 3C and Fig. 5C).

3.2: The quantitative proteomics provide a rich dataset documenting extensive mitochondrial remodelling following kDNA loss. However, the functional significance of many of the observed changes remains unclear (for example, increased abundance of TOM, TIM, MICOS complexes, and various mitochondrial carrier proteins). Experimental validation of selected pathways or protein complexes highlighted in the proteomic analysis would strengthen conclusions regarding their adaptive or mechanistic roles.
R3.2: In terms of a functional assay, we've now included data relating to mitochondrial membrane potential (see R3.1 above). We're unsure if the reviewer is also suggesting western blotting or similar as 'validation' here. If so, we would say that high-throughput quantitative proteomics is typically more robust than antibody-based approaches, which are semi-quantitative at best. This is particularly true when quantifying multiple components of protein complexes. Indeed, we used "high resolution quantitative proteomics on an Orbitrap Astral mass spectrometer with data-independent acquisition" which offers state-of-the art accuracy, sensitivity and depth-of-coverage. See Mehta *et al*., 2022 (PMID: 36456804) for a perspective on this point. Apologies, if western blots are not being suggested here.

3.3: The growth defect observed in kDNA-negative mutants is noted but not analysed further. Additional characterization of this phenotype could provide important insight into the biological costs associated with kDNA loss and help contextualize the adaptive significance of these mutations.
R3.3: We were unclear as to what is being requested here. We have adjusted the text relating to the growth defect, now stating "doubling time increased by approximately 1.5-fold to 9.4 h +/-1.5 h (n = 4); parent doubling time was 6.4 h".

Part III – Minor Issues: Editorial and Data Presentation Modifications

Reviewer 1: (No Response)

Reviewer 2:
2.1mi: Please define kinetoplast more clearly. In some cases it sounds as if this is a separate organelle, in other instances it is stated "loss of the kinetopalst" – so loss of an organelle or loss of the DNA? Throughout the manuscript there are instances where kineoplast loss is used and other where kDNA loss is preferred.
R2.1mi: Good point. We've adjusted several instances of 'kinetoplast' to 'kDNA'.

2.2mi: eliminate use of "approx."
R2.2mi: We are unsure whether this aligns with *PLoS* house style or not, but three instances of "approx." have been changed to "approximately".

2.3mi: Discussion of multidrug resistant – please be more precise by indicating which two classes of drugs are involved with the resistance, or are these parasites resistant to all drugs in use for treatments?
R2.3mi: We've now specified "… to both ATPase and kDNA-targeting drugs; oligomycin and acriflavine, respectively".

2.4mi: abbreviation for kinetoplast DNA is defined in Introduction – it is only needed once but is reintroduced several times.
R2.4mi: We replaced three instances of "kinetoplast DNA" with "kDNA".

2.5mi: Please state the doubling times of the different clones that were generated.
R2.5mi: We now state "increased by approximately 1.5-fold to 9.4 h +/-1.5 h" (also see R3.4mi below).

2.6mi: Authors should acknowledge TritrypDB for information they access – you reference the database in figure 5 legend – full acknowledgment is appropriate.
R2.6mi: Good point. We now cite Shanmugasundram *et al*., 2023 in the Methods section and Fig. 6 legend.

2.7mi: Figure 1D – the authors indicate that there are predicted conformational changes due to the precision editing mutants. Two are obvious a third is less obvious. Could the authors provide additional text to describe the difference since it is hard to see this in a status 2D image.
R2.7mi: We've added "… and more clearly apparent in the $A^{273}P$ mutant" here.

2.8mi: Could the authors link the conformational changes to M282F proteomic outcomes – are there similarities/differences that arise from the L262P and A273P structural changes when compared the current dataset for M282F? Reviewer recognizes that this data might not have been collected in the previous studies and does not think this type of additional proteomics studies of the other mutants are warranted in the current study.
R2.8mi: Proteomics data were not collected in the previous studies.

2.9mi: awkward wording that described which strain was used for sequencing – just state the strain.
R2.9mi: We say we "subjected wild-type cells, kDNA positive $M^{282}F^{TTT}$ edited cells, and all four kDNA negative clones, to whole genome sequencing" so we are unsure what can be usefully adjusted here.

2.10mi: End of paragraph related to Fig3 – statements was…. Thus, proteomic analysis revealed highly specific depletion of subunits of the FO component of the T. b. brucei ATP synthase in homozygous M282FTTT mutants pre kDNA loss. Perhaps it would be better to state it as prior to acriflavine treatment?
R2.10mi: This text is now followed with new detail associated with new Fig. 3 panels B-C, which describe retention of kDNA in these cells and a MitoTracker staining assay. Rather than stating "prior to acriflavine treatment" here, we introduce acriflavine treatment in the following paragraph.

2.11mi: Could the authors report how long it took to achieve stable populations following the sublethal treatment with acriflavine and the dilution cloning process – they mentioned slower doubling times….were the cells considered fully recovered after dilution cloning?
R2.11mi: Yes. These cells were analysed immediately after cloning. We've added "when sufficient cells were available after 7-8 days" to clarify.

Comments on Figure and Legends:
2.12mi: Fig1A: - Nice diagram outlining approach, however in the flask designated oligomycin-resistant cell, why are all the cells blue. As diagramed the approach should yield red and green resistant cells from the precision editing.
R2.12mi: The schematic illustrates introduction of different codons in the library (there are 64 alternatives) and survival of a limited number of these following selection. We've changed the colour to red in the right-hand flask to match the colour in Fig. 1B.

2.13mi: Fig1B; what does the red outline square represent?
R2.13mi: Apologies for this omission. We've now added "(red outline)" in the legend which indicates "the previously reported $M^{282}L$ mutation".

2.14mi: Fig1D: what does the vertical line represent in the alphafold portion of the figure?
R2.14mi: This was erroneously introduced during pdf conversion – now removed.

2.15mi: Fig4A: authors should outline the cells or provide phase images. Current images look as if the nucleus is outside of the cell body.
R2.15mi: We now provide new data in Fig. 3B and Fig. 4A with phase images, and super-resolution images in Fig. 5B and Supplementary Fig. 3 with the cells outlined.

2.16mi: Fig4B: the labelling of the Mini sequence data is a little misleading – is their nomenclature that can be added to indicate those are indeed different classes of sequences?
R2.16mi: We've added ID numbers for each minicircle to clarify.

2.17mi: Fig4B legend: please specify exactly which clone was used for the sequence analysis.
R2.17mi: We've added "kDNA negative clone numbers are indicated in each panel" to the Figure legend and details in the Figure.

2.18mi: Fig. 4C: it is very difficult to see data because of the grey scale chosen. Please improve by using a colored scale. It looks like there might be some maxicircle sequence retained in one of the clones???
R2.18mi: We now use a colour scale. There was one read mapped to the maxicircle sequence in one of the clones, but this was likely a mapping artefact.

2.19mi: Fig5E: The boxes and whiskers are very difficult to see in the current figure.
R2.19mi: We've adjusted the Figure to make the boxes and whiskers more clearly visible.

2.20mi: Figure5E legend: indicate what the two colors represent.
R2.20mi: We've added "Data are shown for kDNA negative clones 1.2 (blue) and 2.1 (red)".

Reviewer 3:
3.1mi: The title appears more suitable for a review article than for a primary research paper. In particular, the term "origins" may imply a broad evolutionary origin of organisms lacking mitochondrial DNA, whereas this study focuses on the identification of three γATPase mutations and experimental validation of one of them. Similarly, the term "consequences" may be somewhat overstated. The consequences described here are largely inferred from proteomic datasets. While valuable, these data provide limited direct insight into how cells functionally adapt to the loss of mitochondrial DNA.
R3.1mi: We've adjusted our title to be more specific and to better reflect our findings: "Genetic origins and proteomic consequences of kinetoplast loss in trypanosomes".

3.2mi: The authors state that the homozygous M282F mutant is resistant to acriflavine, yet kDNA loss is induced using this same drug. Given that similar EC_{50} values are observed for heterozygous L262P and A273P mutants, it appears that this is how "resistance" is defined in this context. However, acriflavine is then used to induce kDNA loss in an acriflavine-resistant strain, which is difficult to reconcile. Clarification of the definition of "resistance" would therefore be helpful. In addition, it would be informative to indicate the acriflavine sensitivity of naturally occurring T. evansi and T. equiperdum, which are expected to be kDNA-independent.
R3.2mi: We are able to induce kDNA loss in these cells because they are acriflavine-resistant, and wild-type cells are acriflavine sensitive because the kDNA is required for viability, but we can see how a misunderstanding can arise here. We've added "a DNA-intercalating agent" and "acriflavine inhibits kDNA replication and segregation" which we hope helps to clarify. We

are not aware of studies that have assessed acriflavine sensitivity in *T. evansi* or *T. equiperdum* isolates.

3.3mi: Can the whole-genome sequencing data be used to assess whether compensatory mutations arise in the nuclear genome following kDNA loss? A brief comment on this possibility would strengthen the manuscript.
R3.3mi: "Acriflavine resistance displayed by homozygous γATPase $M^{282}F^{TTT}$ edited parasites suggested that kDNA would be dispensable in these cells". Consistent with this view, we saw no bottleneck or substantial growth defect during growth in acriflavine. We, therefore, consider compensatory mutations unlikely in the populations analysed here and have adjusted the text to clarify; "sub-EC_{50} dose of acriflavine for 7 days …, which yielded populations containing approximately 50 % kDNA negative cells".

3.4mi: The statement that doubling time increased by 47% is somewhat abstract. Reporting the actual doubling times and stating that growth is slowed approximately 1.5-fold would be clearer and more intuitive.
R3.4mi: We now state "increased by approximately 1.5-fold to 9.4 h +/-1.5 h".

3.5mi: The strain labels used in the supplementary tables (e.g. S22, S24, S312, S315) do not clearly correspond to the strain descriptions in the main text or figures, which use +kDNA and −kDNA designations. Clarification of this nomenclature is needed.
R3.5mi: Thank-you for spotting this discrepancy – now corrected.

3.6mi: Which specific mitochondrial carrier proteins are upregulated? Can any functional adaptations be inferred from these changes?
R3.6mi: Among the MCPs, only MCP14 (Tb927.10.13120) achieved FDR <0.01 in all four -kDNA proteomes. It has been suggested that MCP14 may be involved in proline metabolism, but we prefer not to speculate further here. We have now added the cohort of MCPs to Fig. 6D.

3.7mi: Several sections refer to decreased mitochondrial membrane potential in −kDNA cells. Can this be experimentally measured to directly support this conclusion?
R3.7mi: We've now added data showing experimental measures of mitochondrial membrane potential (see R1.2 above).

3.8mi: The depletion of ATP synthase Fo components prior to kDNA loss is a key observation. Is this more likely due to protein instability (e.g. degradation of unassembled subunits) or to regulated changes in gene expression? Clarification or discussion of this point would be helpful.
R3.8mi: We've now added "possibly due to increased turnover when unassembled" in the Discussion.

3.9mi: The authors suggest that F_1 remains attached to the c-ring in the absence of other Fo components. Could this be tested experimentally, for example using blue native PAGE to assess ATP synthase subcomplexes?
R3.9mi: See R1.3 above.
